# A protein-trap allele reveals roles for *Drosophila* ATF4 in photoreceptor degeneration, oogenesis and wing development

**DOI:** 10.1242/dmm.049119

**Published:** 2022-03-16

**Authors:** Deepika Vasudevan, Hidetaka Katow, Huai-Wei Huang, Grace Tang, Hyung Don Ryoo

**Affiliations:** 1 Department of Cell Biology, New York University Grossman School of Medicine, New York, NY 10016, USA; 2 Department of Cell Biology, University of Pittsburgh School of Medicine, Pittsburgh, PA 15213, USA

**Keywords:** ATF4, Crc, ER stress, Integrated stress response, Retinitis pigmentosa, adRP, Retinal degeneration

## Abstract

Metazoans have evolved various quality control mechanisms to cope with cellular stress inflicted by external and physiological conditions. ATF4 is a major effector of the integrated stress response, an evolutionarily conserved pathway that mediates adaptation to various cellular stressors. Loss of function of *Drosophila* ATF4, encoded by the gene *cryptocephal* (*crc*), results in lethality during pupal development. The roles of *crc* in *Drosophila* disease models and in adult tissue homeostasis thus remain poorly understood. Here, we report that a protein-trap Minos-mediated integration cassette insertion in the *crc* locus generates a Crc-GFP fusion protein that allows visualization of Crc activity *in vivo*. This allele also acts as a hypomorphic mutant that uncovers previously unknown roles for *crc*. Specifically, the *crc* protein-trap line shows Crc-GFP induction in a *Drosophila* model for retinitis pigmentosa. This *crc* allele renders flies more vulnerable to amino acid deprivation and age-dependent retinal degeneration. These mutants also show defects in wing veins and oocyte maturation. Together, our data reveal previously unknown roles for *crc* in development, cellular homeostasis and photoreceptor survival.

This article has an associated First Person interview with the first author of the paper.

## Abstract (precis)

**Summary:** A protein-trap line for *Drosophila* ATF4, encoded by *cryptocephal*, reveals how this integrated stress response transcription factor responds to physiological stress, and demonstrates its role in *Drosophila* development and retinal degeneration.

## INTRODUCTION

Virtually all organisms have evolved stress response mechanisms to mitigate the impact of homeostatic imbalance. The integrated stress response (ISR) pathway, conserved from yeast to humans, is initiated by a collection of stress-responsive kinases. The ISR pathway has been linked to the etiology of a number of human diseases, including neurodegenerative disorders, diabetes and atherosclerosis, among others (Back et al., 2012; Chan et al., 2016; Ivanova and Orekhov, 2016; Ma et al., 2013). Thus, there is immense interest in identifying specific ISR signaling factors and their roles in these pathologies.

Each ISR kinase responds to a different type of stress: PERK (also known as EIF2AK3), an ER-resident kinase, responds to disruption in endoplasmic reticulum (ER) homeostasis (e.g. misfolding proteins and calcium flux); GCN2 (also known as EIF2AK4), a cytoplasmic kinase, responds to amino acid deprivation; PKR (also known as EIF2AK2), a cytoplasmic kinase, responds to double-stranded RNA; and HRI (also known as EIF2AK1), a cytoplasmic kinase, responds to oxidative stress (Donnelly et al., 2013). More recently, MARK2 has been identified as an additional eIF2α kinase that responds to proteotoxic stress (Lu et al., 2021). When activated by the corresponding cellular stress, the ISR kinases phosphorylate the same downstream target: the α-subunit of the initiator methionyl-tRNA (Met-tRNA_i_^Met^) carrying complex, eIF2. Such phosphorylation of eIF2α leads to decreased availability of Met-tRNA_i_^Met^, resulting in lowered cellular translation (Sonenberg and Hinnebusch, 2009). However, the translation of some mRNAs with unusual 5′ leader arrangements, such as the one encoding the ISR transcription factor ATF4, is induced even under such inhibitory conditions (Hinnebusch et al., 2016). ATF4 is a bZIP (basic leucine zipper) transcription factor that induces the expression of stress response genes, including those involved in protein folding chaperones, amino acid transporters and antioxidant genes (Back et al., 2009; Fusakio et al., 2016; Han et al., 2013; Shan et al., 2016).

The number of ISR kinases varies depending on organismal complexity, e.g. GCN2 in *Saccharomyces cerevisiae* (yeast), GCN2 and PERK in *Caenorhabditis elegans* (worms) and *Drosophila melanogaster* (flies), and additional ISR kinases in *Danio rerio* (zebrafish) and other vertebrates (Mitra and Ryoo, 2019; Ryoo, 2015). Although these kinases induce a few downstream transcription factors (Andreev et al., 2015; Brown et al., 2021; Palam et al., 2011; You et al., 2021), ATF4 remains the best characterized (Donnelly et al., 2013). *Drosophila* has a functionally conserved ortholog referred to as *cryptocephal* (*crc*) (Fristrom, 1965; Hewes et al., 2000). In addition to its well-characterized roles during cellular stress, a plethora of studies have demonstrated roles for ISR signaling components during organismal development (Mitra and Ryoo, 2019; Pakos-Zebrucka et al., 2016). In *Drosophila*, loss of *Gcn2* results in decreased lifespan and increased susceptibility to amino acid deprivation and bacterial infection (Kang et al., 2017; Vasudevan et al., 2017). *Drosophila Perk* is highly expressed in the endodermal cells of the gut during embryogenesis, and has also been demonstrated to regulate intestinal stem cells in adults (Wang et al., 2015). Although both *Gcn2* and *Perk Drosophila* mutants survive to adulthood (Kang et al., 2017; Vasudevan et al., 2020), mutations in *crc* result in significant lethality during larval stages. The *crc* hypomorphic point mutant *crc^1^*, which causes a single amino acid change, results in delayed larval development and subsequent pupal lethality (Fristrom, 1965; Hewes et al., 2000; Vasudevan et al., 2020). The most striking phenotype of the *crc^1^* mutants is the failure to evert the adult head during pupariation, along with failure to elongate their wings and legs (Fristrom, 1965; Gauthier et al., 2012; Hewes et al., 2000; Vasudevan et al., 2020).

The larval and pupal lethality of known *crc* alleles have limited our understanding of its role in adult tissues. Additionally, study of the role of Crc using mitotic clones has been impeded by the cytogenetic proximity of *crc* to the widely used flippase recognition target (FRT)40 element. Here, we report that a GFP protein-trap reporter allele in the *crc* locus acts as a hypomorphic mutant that survives to adulthood. We use this allele to discover that loss of *crc* results in higher rates of retinal degeneration in a *Drosophila* model of autosomal dominant retinitis pigmentosa (adRP), a human disease with an etiology linked to ER stress. Adult *crc* mutants show increased susceptibility to amino acid deprivation, consistent with what was previously known for GCN2. Additionally, we observe several developmental defects in adult tissues, including reduced female fertility due to a block in oogenesis. We also observe wing vein defects and overall reduced wing size in both male and female *crc* mutants.

## RESULTS

### *crc^GFSTF^* is a faithful reporter for endogenous Crc levels

**Fig. 1.**
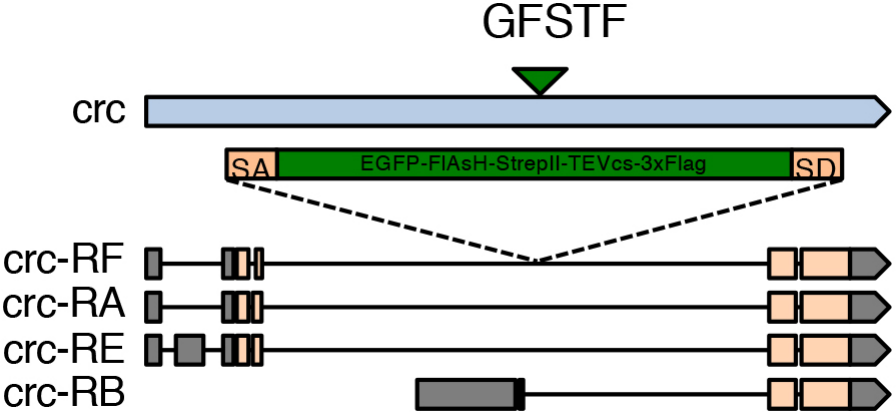
**Schematic of the *crc* cytogenetic locus.** The *crc* gene (blue bar) is known to encode at least four splice variants, *crc-RA*, *-RB*, *-RE*, and *–RF.* These splice isoforms vary in their 5′ leader sequences (gray bars) and their coding exons (beige bars). MiMIC-mediated insertion of the GFSTF cassette in the genomic locus (green triangle) with splice acceptor (SA) and splice donor (SD) sequences predicts the inclusion of a multi-tag exon (green box) in all the *crc* isoforms except *crc-RB*.

In seeking endogenous reporters of Crc activity, we examined a ‘protein trap’ line for *crc,* generated as part of the Gene Disruption Project (Nagarkar-Jaiswal et al., 2015a,b; Venken et al., 2011). The Gene Disruption Project is based on a Minos-mediated integration cassette (MiMIC) element inserted randomly into various regions in the *Drosophila* genome. The cassette can be subsequently replaced with an EGFP-FlAsH-StrepII-TEV-3xFlag (GFSTF) multi-tag cassette using recombination-mediated cassette exchange. One such insertion recovered through this project is in the intronic region of the *Drosophila crc* locus, which has been subsequently replaced with an GFSTF multi-tag cassette (Fig. 1). The splice donor and acceptor sequences flanking the cassette ensure that the GFSTF multi-tag is incorporated in the coding sequence of most *crc* splice isoforms to generate a Crc fusion protein (Fig. 1). This *crc* reporter allele is henceforth referred to as *crc^GFSTF^*, with the encoded fusion protein referred to as Crc-GFP.

**Fig. 2.**
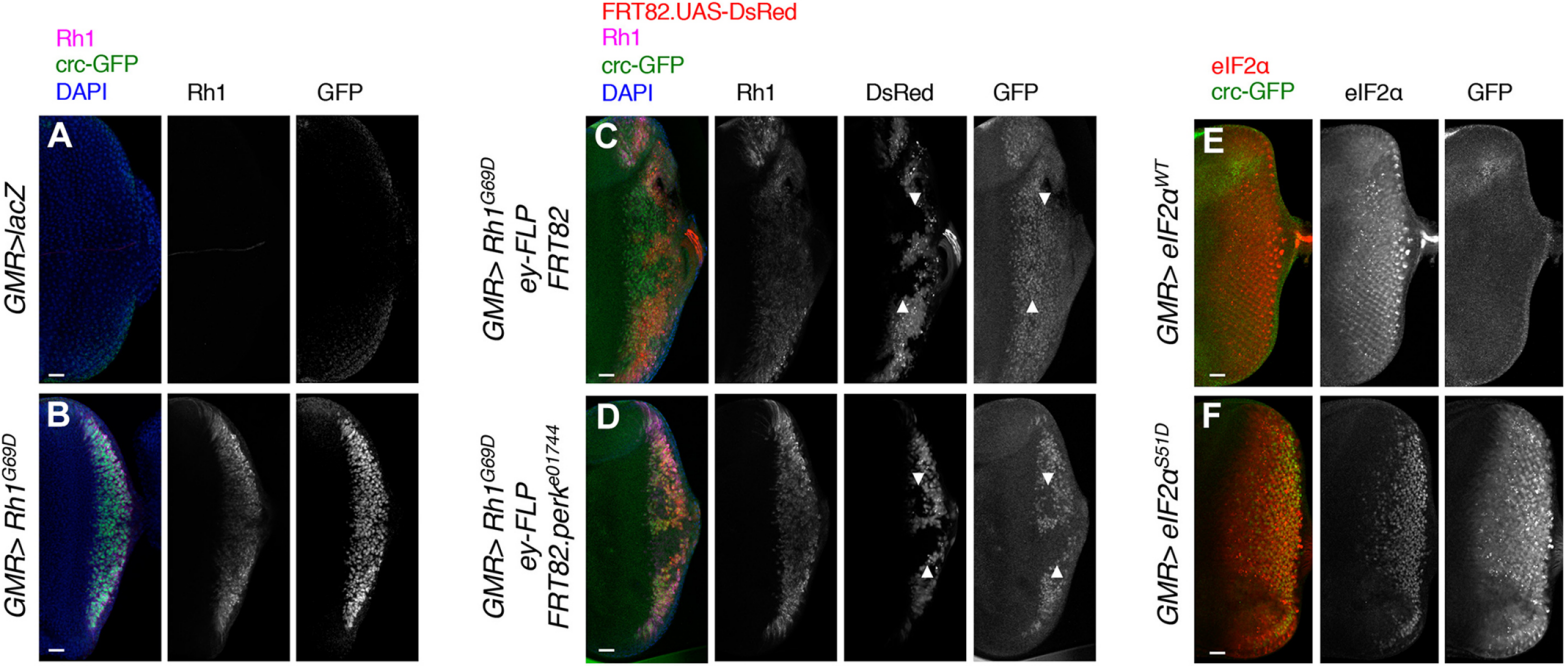
***crc^GFSTF^* is a reporter for Crc activity *in vivo*.** (A,B). Confocal images from eye imaginal discs from wandering third instar larva in which *GMR-Gal4* drives the expression of either a control protein (*GMR>lacZ*) or mutant Rh1 (*GMR>Rh1^G69D^*), in the *crc^GFSTF^/^+^* background. Here and in following images, the Crc-GFP fusion protein was detected with anti-GFP (green), Rh1 was detected with 4C5 antibody (magenta) and DAPI (blue) stains the nucleus. Anterior is the left, and posterior to the right. (C,D) Confocal images of eye imaginal discs misexpressing Rh1^G69D^ (*GMR>Rh1^G69D^*) showing control clones (C, FRT82) and *Perk* mutant clones (D, *FRT82.perk^e01744^*) generated by eyeless-flippase (*ey-FLP*) in the *crc^GFSTF^/^+^* background. Clones are negatively marked with DsRed (red), also driven by *GMR>* (white arrowheads), and demonstrate the effect of loss of *Perk* on Crc-GFP induction in response to Rh1^G69D^. (E,F) Confocal images of eye imaginal discs showing Crc-GFP expression in response to wild-type eIF2α (*eIF2α^WT^*) or phospho-mimetic eIF2α (*eIF2α^S51D^*) ectopically expressed with *GMR-Gal4* (*GMR>*). Ectopic expression was confirmed by staining with anti-eIF2α (red). Scale bars: 25 µm.

Our laboratory and others have utilized acute misexpression of Rh1^G69D^, an ER stress-imposing mutant protein, in third instar larval eye disc tissues, using a *GMR-Gal4* driver (*GMR>Rh1^G69D^*), as a facile method to activate the *Perk-crc* pathway (Kang et al., 2015, 2017; Ryoo et al., 2007). We tested the utility of the *crc^GFSTF^* allele as an endogenous reporter for Crc levels, and found robust induction of Crc-GFP in third instar larval eye discs in response to misexpression of Rh1^G69D^ protein, but not in response to control lacZ protein in the *crc^GFSTF^*/*^+^* background (Fig. 2A,B). To validate that such induction was downstream of PERK activation, caused by the misexpression of Rh1^G69D^, we generated *Perk* mutant FRT clones negatively marked by DsRed expression in the glass multiple reporter (GMR) compartment using ey-FLP. Although control clones showed no change in induction of Crc-GFP (Fig. 2C), *Perk^e01744^* mutant clones showed a complete loss of Crc-GFP in *GMR>Rh1^G69D^* eye imaginal discs (Fig. 2D). We also validated these observations in whole-animal *Perk^e01744^* mutants, in which we observed a complete loss of Crc-GFP in *GMR>Rh1^G69D^* eye imaginal discs (Fig. S1).

To examine whether Crc-GFP could also be used as a readout for GCN2 activation, we dissected fat bodies from wandering third instar larva, in which we have previously reported GCN2-dependent Crc activation (Kang et al., 2015, 2017). We observed Crc-GFP signal localized to the nucleus in the larval fat bodies (Fig. S2). Such signal was substantially depleted using fat body-specific RNAi knockdown of ATF4 or GCN2 (Fig. S2).

As the induction of Crc in response to stress is regulated at the level of mRNA translation, we wanted to ensure that the induction of Crc-GFP we observed in Fig. 2A-D is reflective of translation regulation via the *crc* 5′ leader sequence. The *crc* 5′ leader is structured such that the main open reading frame of *crc* is favorably translated when eIF2 availability is reduced, such as during phospho-inactivation of eIF2α by ISR kinases (Hinnebusch, 1984; Kang et al., 2015). It has also been previously demonstrated that phosphorylation of a subset of cellular eIF2α is sufficient to diminish initiator methionine availability, thus mimicking ISR activation (Ramaiah et al., 1994). To imitate reduction of eIF2 availability by ISR kinases, we generated a phospho-mimetic transgenic line in which the Ser51 in eIF2α is mutated to Asp51 (*UAS-eIF2α^S51D^*). We also generated a corresponding control transgenic line containing wild-type eIF2α (*UAS-eIF2α^WT^*). We used this genetic mimetic of ISR activation to test whether *crc^GFSTF^* reported *crc* translation induction under reduced eIF2 availability conditions. While *GMR>eIF2α^WT^* discs showed no detectable levels of Crc-GFP, we found that *GMR>eIF2α^S51D^* led to robust induction of Crc-GFP in eye discs, as detected by immunostaining with anti-GFP (Fig. 2E,F). These data demonstrate the applicability of *crc^GFSTF^* as a reliable reporter of endogenous Crc expression downstream of ISR activation.

### *crc^GFSTF^* is a hypomorphic *crc* mutant allele

**Fig. 3.**
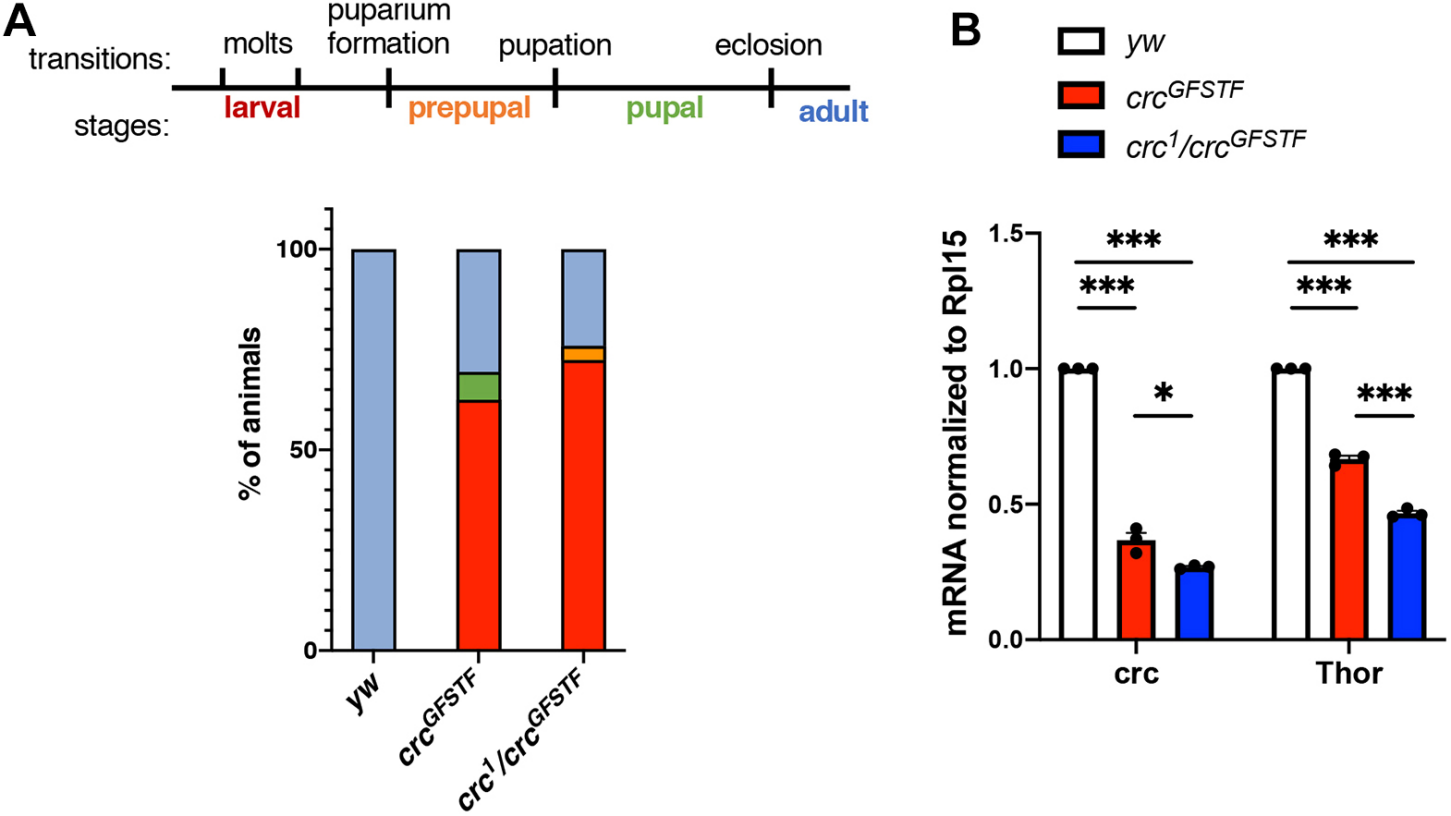
***crc^GFSTF^* is a *crc* hypomorphic allele.** (A) Top: schematic showing transitions and stages during *Drosophila* development. Bottom: lethal phase analysis for control (*yw*), *crc^GFSTF^* homozygotes and transheterozygotes (*crc^GFSTF^/crc^1^*), color-coded per the schematic. *n*=100 for each genotype. *P*<0.0001 [χ^2^ test done pair-wise between the control (*yw*) and the mutants]. (B) qPCR analysis of *crc* and its transcriptional target *Thor*, normalized to *Rpl15* from wandering third instar larval stages when Crc expression is known to be elevated. Data represent the mean of three independent experiments and are mean±s.e.m. *P<0.05, ***P<0.0001 (paired two-tailed Student's *t*-test).

Similar to the previously characterized *crc* hypomorphic mutant allele *crc^1^* (Fristrom, 1965; Hewes et al., 2000), we observed that flies homozygous for *crc^GFSTF^* exhibited a delay in head eversion and showed anterior spiracle defects. However, unlike the *crc^1^* mutants, none of the *crc^GFSTF^* homozygotic pupae exhibited complete loss of head eversion, indicating that *crc^GFSTF^* is likely a weaker hypomorphic allele than *crc^1^*. To further assess the effects of the *crc^GFSTF^* allele, we performed lethal phase analysis of development, starting at the first instar larva. We found that a little over 50% of *crc^GFSTF^* homozygotes were larval lethal (Fig. 3A), which is remarkably similar to the larval lethality we previously reported for *crc^1^* (Vasudevan et al., 2020). However, unlike *crc^1^* homozygotes, only a small percentage of *crc^GFSTF^* homozygotes showed prepupal and pupal lethality, with ∼30% of animals eclosing as adults (Fig. 3A). To ensure that these developmental defects cannot be attributed to background mutations in *crc^GFSTF^*, we performed lethal phase analysis on *crc^GFSTF^* in transheterozygotic combinations with the hypomorphic *crc^1^* allele. We found that *crc^GFSTF^/crc^1^* transheterozygotes showed similar levels of larval and pupal lethality to *crc^GFSTF^* homozygotes, with ∼25% of animals surviving to adulthood (Fig. 3A). These data together suggested that the *crc^GFSTF^* allele may function as a *crc* loss-of-function allele.

To examine whether *crc* transcript levels are affected in *crc^GFSTF^* mutants, we performed qPCR in the wandering third instar larval stage, when Crc activity is known to be high in fat tissues (Kang et al., 2015, 2017). We found that *crc^GFSTF^* homozygotes showed a ∼65% decrease in *crc* transcript levels in comparison to control animals (Fig. 3B). We also tested Crc activity by measuring mRNA levels of the well-characterized Crc transcriptional target *4E-BP* (*Drosophila Thor*). We observed ∼40% lower levels of *Thor* in *crc^GFSTF^* in comparison to control animals (Fig. 3B). This reduction in transcript levels of Crc targets was also reproducible in *crc^GFSTF^*/*crc^1^* transheterozygotes (Fig. 3B). Taken together, these data indicate that *crc^GFSTF^* acts as a mild hypomorphic mutant allele of *crc*.

### *crc* has a protective role in age-related retinal degeneration and amino acid deprivation

Nearly 30% of all adRP mutations are found in the rhodopsin gene (Illing et al., 2002; Kaushal and Khorana, 1994). Several of these Rhodopsin mutations result in misfolding proteins, which inflict ER stress (Kroeger et al., 2019). However, the role of ATF4 in adRP has remained unclear. We sought to resolve this using the *crc^GFSTF^* allele in a *Drosophila* model of adRP.

Clinically, adRP is characterized by age-related loss of peripheral vision, resulting in ‘tunnel vision’ and night blindness, due to degeneration of rod photoreceptors (Kaushal and Khorana, 1994). The *Drosophila* genome encodes several Rhodopsin genes, including *ninaE,* which encodes the Rhodopsin-1 (Rh1) protein. The *ninaE^G69D^* mutation captures essential features of adRP etiology: flies bearing one copy of the dominant *ninaE^G69D^* allele exhibit age-related retinal degeneration, as seen by photoreceptor cell death (Colley et al., 1995; Kurada and O'Tousa, 1995). This mutant encodes a protein with a negatively charged Asp residue in the transmembrane domain; therefore, it is predicted to disrupt the folding properties of Rh1. Consistently, the *ninaE^G69D^* mutant activates ER stress markers in photoreceptors (Ryoo et al., 2007). More recent gene expression profiling experiments found that *ninaE^G69D^/*^+^ photoreceptors induce many ISR-associated genes, including *crc* itself (Huang and Ryoo, 2021).

**Fig. 4.**
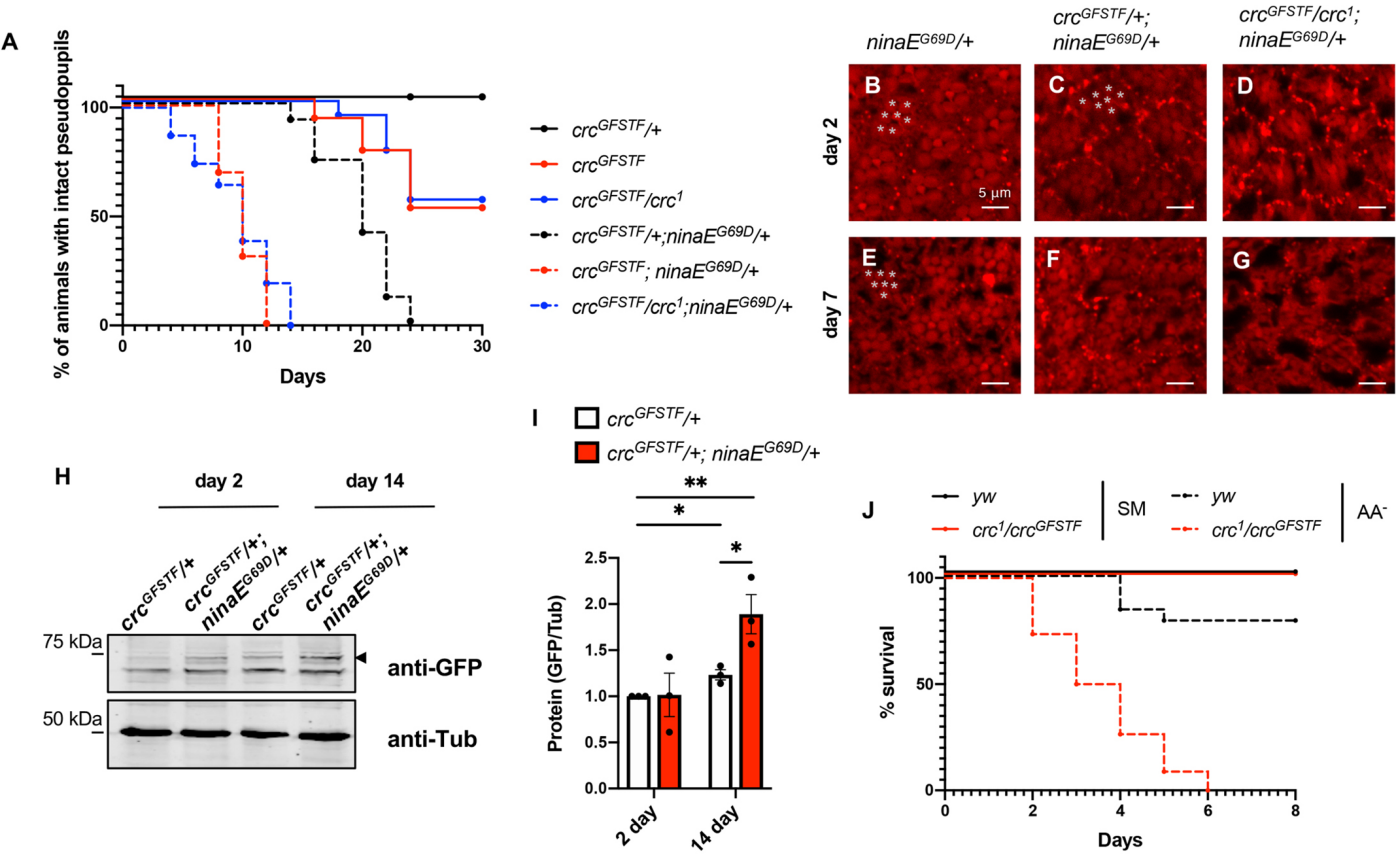
**Crc mediates *Perk* and *Gcn2* phenotypes in adult animals.** (A) Time course of pseudopupil degeneration in control and *ninaE^G69D^*/*^+^* flies in *crc* heterozygote (*crc^GFSTF^*/+) and transheterozygous mutants (*crc^GFSTF^/crc^1^*). The difference in the course of retinal degeneration between the following pairs is statistically significant, as assessed by the log-rank (Mantel–Cox) test (*P*<0.001): *crc^GFSTF^*/*^+^* and *crc^GFSTF^/*^+^;*ninaE^G69D^/^+^*, *crc^GFSTF^*/*crc^1^* and *crc^GFSTF^/crc^1^*;*ninaE^G69D^/^+^,* and, *crc^GFSTF^*/^+^ and *crc^GFSTF^/crc^1^*. (*n*=100). (B-G) Confocal images of retinae dissected from young (2 days old) and older (7 days old) adult flies of indicated genotypes, stained with phalloidin (red) to mark actin in photoreceptors. Each individual ommatidium comprises eight photoreceptors (R1-R8), with only seven visible in this projection (marked by white stars). (H) Western blot analysis of fly head extracts from young (1-2 days) and aged (14-16 days) control flies and *ninaE^G69D^*/*^+^* animals also heterozygous for *crc^GFSTF^*. Upper panel shows the blot probed with anti-GFP to detect the Crc-GFP fusion protein (distinguished by the black arrowhead), and the lower panel shows Tubulin (anti-Tub) as a loading control. (I) Quantification of western blotting data in B showing Crc-GFP normalized to Tubulin. Data are mean±s.e.m. from three independent experiments. **P<0.001, *P<0.01 (paired two-tailed Student's *t*-test). (J) Time course of survival rate of adult females of indicated genotypes when fed with standard medium (SM, solid lines) or amino acid-deprived medium (AA^−^, broken lines). The curves for the flies fed standard medium for *yw* (solid black) and *crc^GFSTF^/crc^1^* (solid red) overlap entirely. The difference in the survival rates between the following pairs is statistically significant as assessed by the log-rank (Mantel–Cox) test (*P*<0.001): *yw* (SM) and *yw* (AA^−^), *crc^GFSTF^/crc^1^* (SM) and *crc^GFSTF^/crc^1^* (AA^−^), and *yw* (AA^−^) and *crc^GFSTF^/crc^1^* (AA^−^). (*n*=100).

We found that *crc^GFSTF^*/*crc^1^; ninaE^G69D^/^+^* animals exhibited rapid retinal degeneration in comparison to *crc^GFSTF^*/*^+^; ninaE^G69D^/^+^* control animals, as monitored by pseudopupil structures in live flies over a time course of 30 days (Fig. 4A). The earliest timepoint when control animals exhibit retinal degeneration is typically around 13-15 days; however, *crc* homozygous mutant animals exhibited onset of retinal degeneration as early as 4 days, with all animals displaying complete loss of pseudopupil structures by day 14 (Fig. 4A). Further analysis of photoreceptor integrity by actin immunostaining following dissection showed that even young (2 days old) *crc^GFSTF^*/*crc^1^; ninaE^G69D^/^+^* flies showed evident disruption of ommatidial organization in comparison to *ninaE^G69D^/^+^* animals, in which ommatidial organization was relatively unperturbed (Fig. 4B-D). At day 7, the majority of *ninaE^G69D^/^+^* animals showed intact pseudopupils and identifiably regular ommatidial arrangements (Fig. 4A,E). In contrast, we observed considerable disruption of ommatidial structures in both *crc^1^* and *crc^GFSTF^*/*crc^1^* mutants bearing the *ninaE^G69D^* allele. (Fig. 4A,F,G). Interestingly, we also found that *crc^GFSTF^*/*crc^1^* animals exhibited age-dependent retinal degeneration even in the absence of *ninaE^G69D^*, indicating that age-related physiological decline requires a protective role for Crc in photoreceptors (Fig. 4A).

To measure the expression of Crc in aging photoreceptors, we performed western blotting of adult fly heads from young (2 days old) and old (14 days old) flies to detect Crc-GFP. Although young control flies (*crc^GFSTF^*/*^+^*) showed very low levels of Crc-GFP, flies bearing one copy of *ninaE^G69D^* showed a substantial induction of Crc-GFP (Fig. 4H,I). We observed that Crc-GFP increases with age in 14-day-old control flies (*crc^GFSTF^*/^+^), with a concomitant increase in Crc-GFP in *ninaE^G69D^/^+^* flies (Fig. 4H,I). These data substantiate the role of Crc in photoreceptors suffering from ER stress induced by misfolding-prone Rh1^G69D^, thus providing a basis for the protective roles of *Perk* in retinal degeneration.

In addition to rendering a protective effect during ER stress inflicted by Rh1^G69D^, we also tested whether Crc had an effect during amino acid deprivation in adult animals. We tested this by subjecting *crc^GFSTF^*/*crc^1^* animals to amino acid deprivation by rearing animals on 5% sucrose-agar. Although a majority of control animals survived up to 8 days, *crc^GFSTF^*/*crc^1^* animals steadily succumbed to amino acid deprivation, starting at day 2 with no survivors by day 6 (Fig. 4J). This is consistent with the idea that Crc mediates the GCN2 response to amino acid deprivation in adult *Drosophila*.

### *crc* mutants show wing size and vein defects

**Fig. 5.**
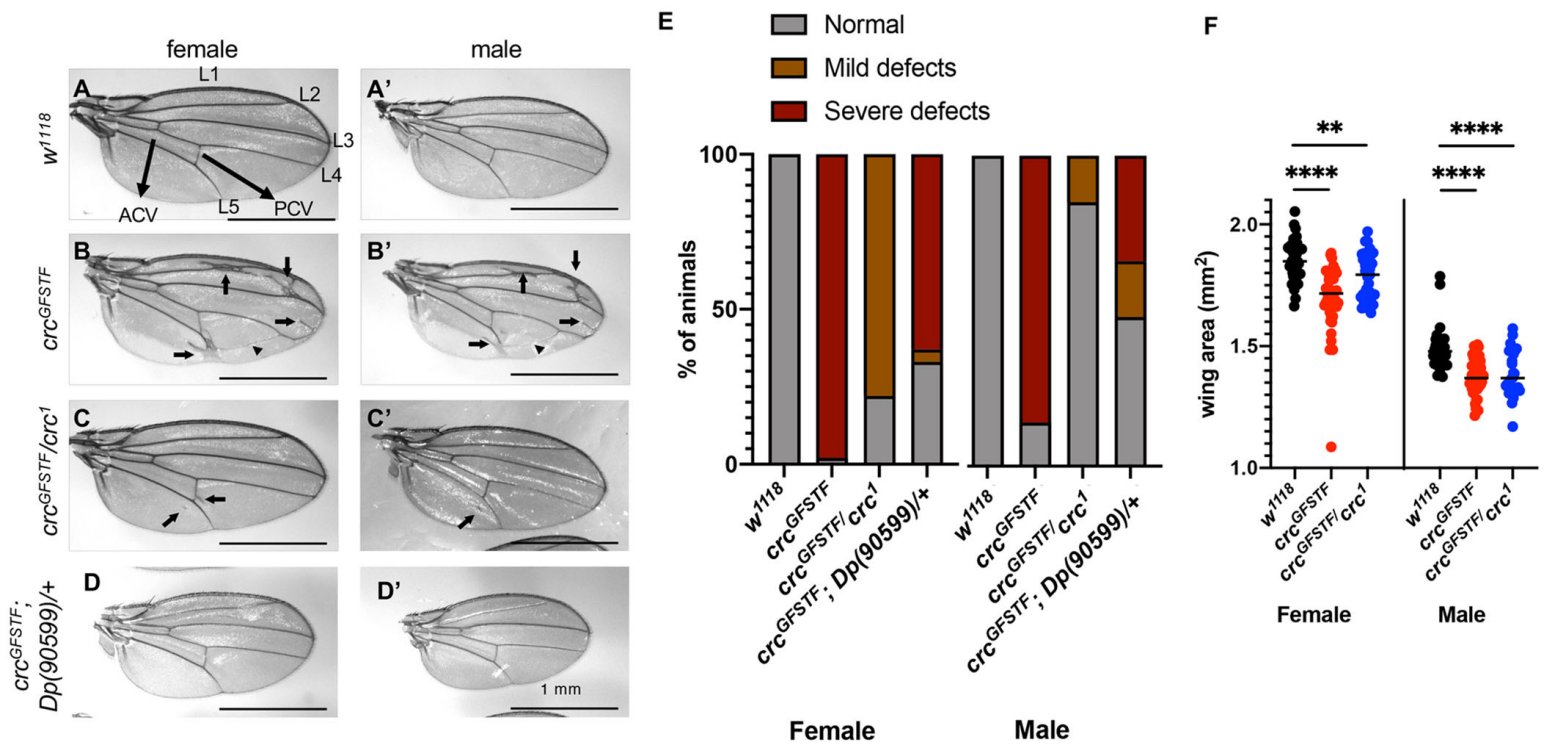
**Adult *crc* mutants display developmental defects in the wing.** (A-D) Grayscale images of the right wing from female (A-D) or male (A′-D′) flies from the indicated genotypes. (A) Arrangement of wing veins in control (*w^1118^*) flies with L1-L5 marking longitudinal veins, and arrows marking the anterior cross vein (ACV) and posterior cross vein (PCV). Ectopic longitudinal veins in *crc^GFSTF^* homozygotes and *crc^GFSTF^/crc^1^* transheterozygotes (B,B′,C,C′) are marked by arrows, and arrowheads point to ectopic cross veins. (D,D′) Rescue of ectopic venation by the introduction of a chromosomal duplication, *Dp (90599)*, spanning *crc*. (E) The severity and penetrance of the ectopic vein phenotype in A-D quantified from 40 animals of each sex of the indicated genotypes. Animals showing venation, such as in B, were classified severe, and animals in C were classified mild. *P*<0.001 [χ^2^ test performed pair-wise between the control (*w1118*) and the mutants]. (F) Area of the right wing from male and female flies of the indicated genotypes as measured in ImageJ. *n*≥27 for each genotype. Data are mean±s.e.m. **P<0.001, ****P<0.00001 (unpaired two-tailed Student's *t*-test).

*crc^GFSTF^* provided an opportunity to examine previously unreported roles for *crc* in adult flies. We first observed that wings from both *crc^GFSTF^* homozygotes and *crc^GFSTF^*/*crc^1^* transheterozygotes showed a range of venation defects (Fig. 5A-C). The *Drosophila* wing has five longitudinal veins (annotated L1-L5) and two cross veins, anterior and posterior, labeled ACV and PCV, respectively (Fig. 5A). Severe wing defects in *crc^GFSTF^* homozygous flies were characterized by ectopic venation on L2, between L3 and L4, on L5, and ectopic cross veins adjacent to the PCV (Fig. 5B,B′). *crc^GFSTF^*/*crc^1^* transheterozygotes largely showed milder wing defects, characterized by ectopic venation on the PCV and on L5 (Fig. 5C,C′). We quantified these wing phenotypes in over 40 animals of each sex, and found that the penetrance and severity of the phenotype was much stronger in females than in males (Fig. 5E). To ensure that the phenotypes were not due to background mutations, we performed a genomic rescue experiment using a BAC-clone based chromosomal duplication covering the *crc* locus, *Dp (90599)*. We found venation phenotypes in *crc^GFSTF^* homozygotic mutants to be substantially, albeit incompletely, rescued by *Dp (90599)* (Fig. 5D-E).

We also observed that *crc* mutant wings were smaller than in control animals (Fig. 5A-C). Quantification of the wing area revealed a statistically significant decrease in wing blade size in *crc^GFSTF^* and *crc^GFSTF^*/*crc^1^* (Fig. 5F). To exclude the possibility of dominant negative effects of *crc^GFSTF^*, we also tested wings from *crc^GFSTF^*/*^+^* heterozygotes but found no wing defects in these animals (Fig. S3). We were unable to detect Crc-GFP expression in the developing wing discs. This may be because of insufficient sensitivity of the reporter, or possibly indicates a non-autonomous role for *crc* in wing development. It is notable that *Gcn2* depletion in the wing reportedly causes venation (Malzer et al., 2018). Thus, our results suggest that *Gcn2*-mediated Crc activation contributes to proper wing vein development.

### *crc* mutants exhibit decreased fertility due to defects in oogenesis

**Fig. 6.**
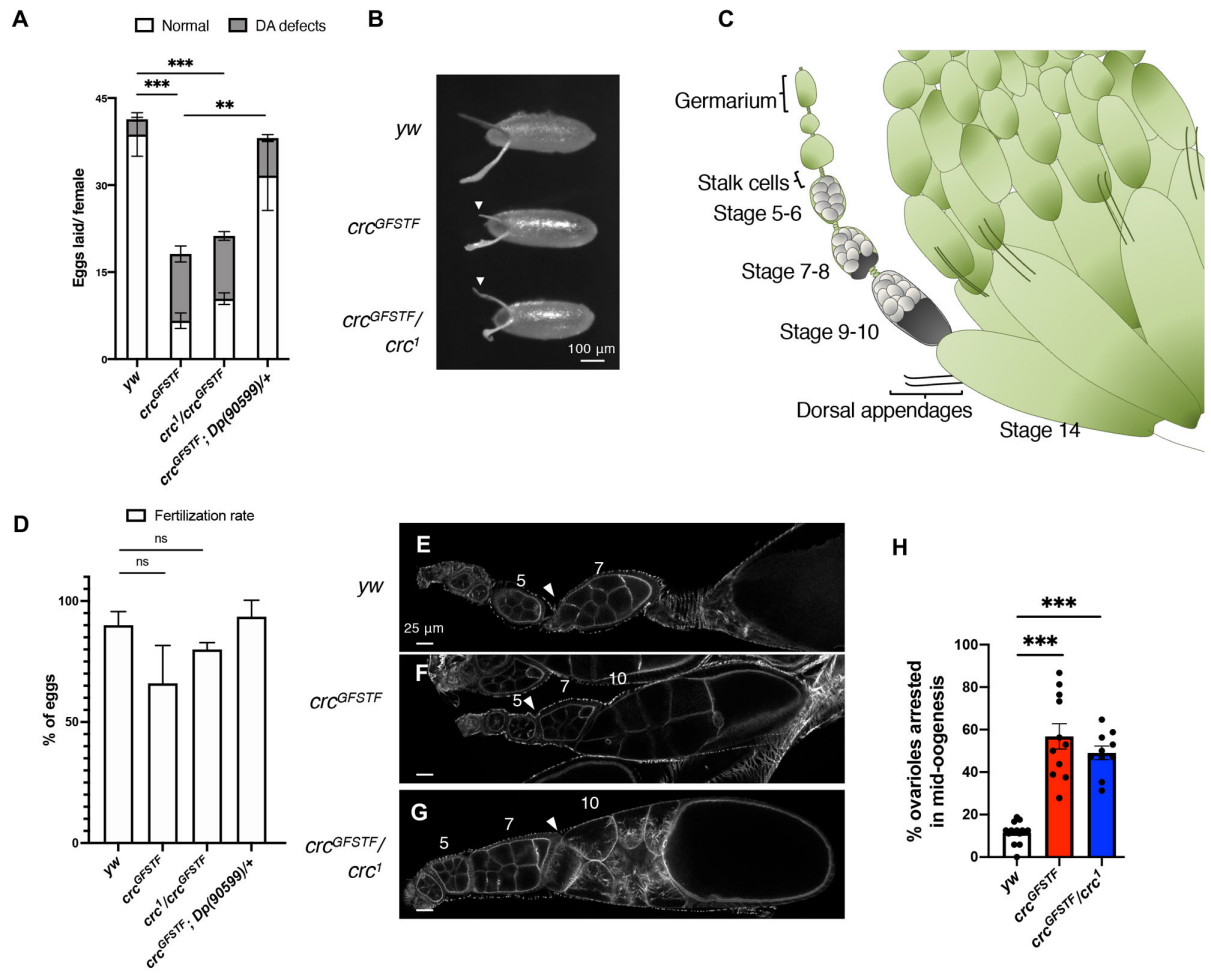
***crc* mutant females show reduced fertility.** (A) Total number of eggs laid per female in a 24-h period for control (*yw*), *crc* mutants and *crc^GFSTF^* rescued with Dp(90599), with the proportion of eggs showing dorsal appendage (DA) defects in gray. (B) Grayscale images of 0-24 h eggs from females of the indicated genotypes. White arrowheads indicate dorsal appendage defects in eggs laid by *crc* mutant females compared to well-formed and elongated dorsal appendages in eggs laid by control females (*yw*). (C) Schematic of ovariole from a partially visible ovary. The stages of the egg chambers can be approximately identified by the arrangement of cells within each ovariole, as indicated in gray, with the oocyte becoming readily visible starting at stage 7. (D) Fertilization rate as indicated by the percentage of dj-GFP^+^ eggs laid by females of the indicated genotype and *Dj-GFP* males. (E-G) Confocal images of individual ovarioles from indicated genotypes, counterstained with phalloidin (actin). Control ovarioles (*yw*) show clearly delineated individual egg chambers (white arrowheads, C) that are appropriately sized for each stage. *crc* mutant ovarioles (*crc^GFSTF^*, *crc^GFSTF^/crc^1^*) show enlarged stage 10 egg chambers, with no clear delineation between individual egg chambers (white arrowheads), indicating a defect in oogenesis. Approximate stages of egg chambers are indicated. (H) Percentage of ovarioles per ovary showing enlarged stage 10 egg chambers, which are indicative of a mid-oogenesis arrest. Data are mean±s.e.m. from four independent experiments with five females per experiment (A), three independent experiments (D), or individual ovaries of 11 animals (H). **P<0.001; ***P<0.0001; ns, not significant (unpaired two-tailed Student's *t*-test).

In trying to establish a stock of *crc^GFSTF^*, we observed that when mated to each other, *crc^GFSTF^* homozygotic males and females produced no viable progeny, with very few of the eggs laid hatching to first instar larvae. To determine whether this loss of fertility in *crc^GFSTF^* is due to loss of fertility in males, females or both, we separately mated *crc* mutant females to healthy control (genotype; *yw*) males and vice versa. We observed that although *crc^GFSTF^* and *crc^GFSTF^*/*crc^1^* males produced viable progeny at similar rates to control *yw* males (data not shown), *crc* mutant females showed ∼50% reduction in egg laying compared to control females (Fig. 6A), again with very few of the eggs laid hatching to first instar larvae. Upon closer observation, we saw defects in the dorsal appendages of eggs laid by *crc* mutant females, from mild phenotypes, such as the shortening of the appendages, to a complete absence of one or both appendages (Fig. 6B). The proportion of eggs showing such dorsal appendage defects were significantly higher in *crc* mutants than in control *yw* animals. Both the overall fertility defect and dorsal appendage defects in *crc* mutants were significantly rescued with the introduction of *Dp(90599)* (Fig. 6A).

Dorsal appendages are specified and develop in the final stage of oogenesis. Each *Drosophila* ovary comprises 14-16 developing follicles called ovarioles, with germline stem cells, residing at the anterior apex, undergoing differentiation along the ovariole in individual egg chambers (Lobell et al., 2017). Each egg chamber represents a distinct stage in ovulation, with stage 14 representing a mature egg (see schematic in Fig. 6C). To examine whether the dorsal appendage defects resulted in decreased fertilization of eggs, we measured fertilization rates of laid eggs by mating control and *crc* mutant females with *don juan-GFP* (*dj-GFP*) males (Santel et al., 1997). Dj-GFP marks individual spermatids, which can be observed in fertilized eggs under a fluorescent microscope. Our analysis showed no significant change in rates of fertilization between eggs laid by *yw, crc^GFSTF^* and *crc^1^/crc^GFSTF^* females (Fig. 6D). These data indicate that the fertility defects in *crc* mutants are due to loss of Crc function in female flies.

To further dissect the fertility defects, we examined ovaries from *crc* mutant animals. We observed that ovaries from *crc^GFSTF^* and *crc^GFSTF^*/*crc^1^* were considerably swollen compared to control ovaries (Fig. S4A). Several ovarioles within *crc* mutant ovaries showed enlarged stage 10 egg chambers, indicative of a stall in oogenesis (white arrowheads in Fig. S4A). Indeed, examination of individual ovarioles from *crc* mutant ovaries counterstained for actin showed that loss of *crc* results in an abnormal arrangement of early stage egg chambers (Fig. 6E,F). Although ovarioles from control animals showed sequentially staged and spaced egg chambers culminating in mature stage 14 eggs (Fig. 6C,E), ovarioles from *crc^GFSTF^* and *crc^GFSTF^*/*crc^1^* appeared to be arrested at stage 10, with improper spacing between egg chambers in earlier stages (white arrowheads, Fig. 6F,G). We quantified the number of ovarioles that displayed such arrest and found that more than half of *crc* mutant ovarioles (∼9) in each ovary showed stage 10 arrest compared to an average of 2-3 ovarioles arrested in ovaries from corresponding control animals (Fig. 6H).

**Fig. 7.**
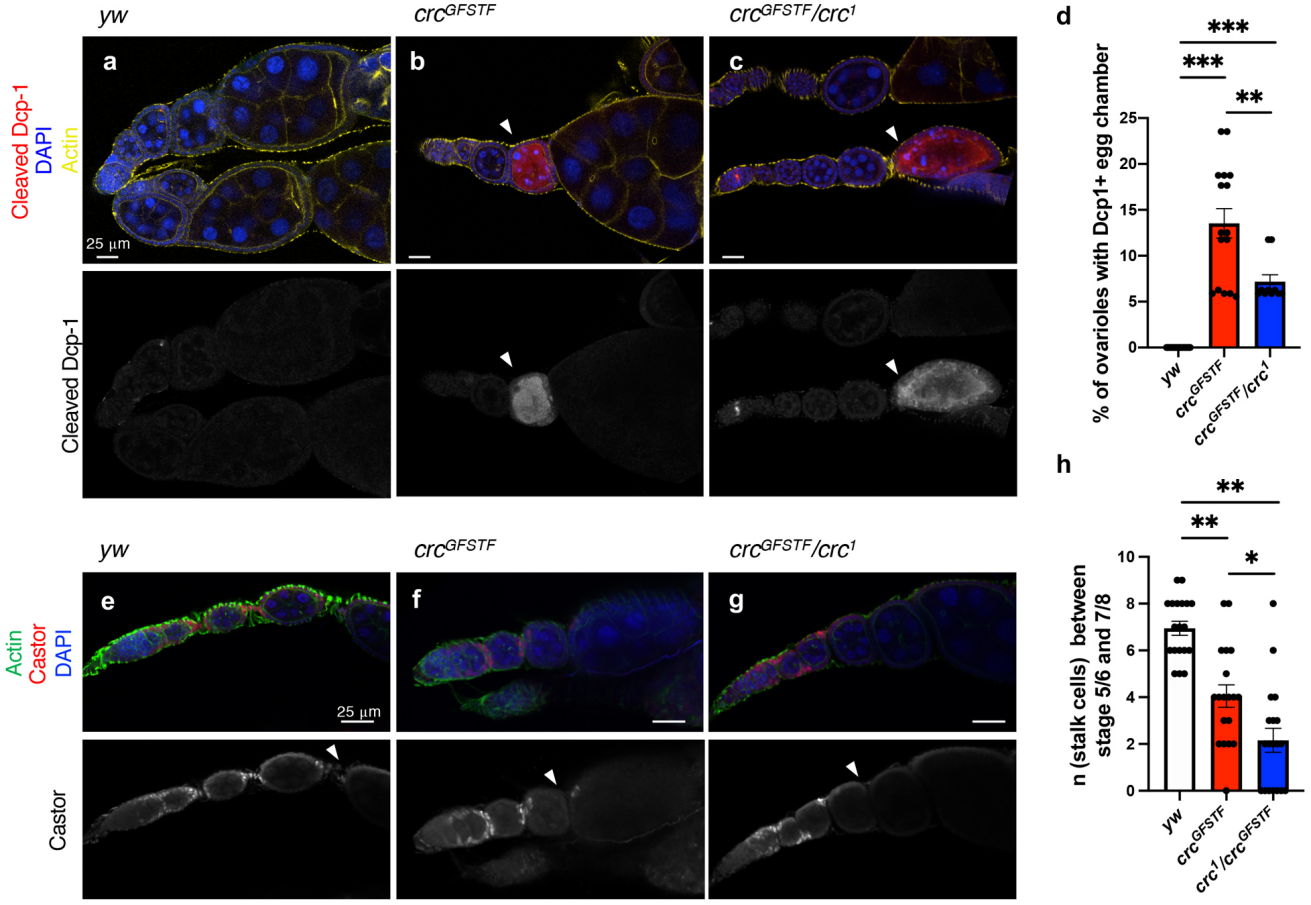
***crc* mutant ovaries show increased egg chamber death and stalk cell dysregulation.** (A-C) Confocal images of ovaries from the indicated genotypes stained with the cell death marker cleaved Dcp-1 (red), nuclei counterstained with DAPI (blue) and phalloidin marking actin (yellow). White arrowheads point to egg chambers in *crc* mutant ovarioles (*crc^GFSTF^*, *crc^GFSTF^/crc^1^*) that show elevated Dcp-1 staining. The lower panels show Dcp-1 only channel. (D) Quantification of the percentage of ovarioles per egg showing cleaved Dcp-1^+^ egg chamber (A-C). (E-G) Confocal images of ovaries from the indicated genotypes stained with the stalk cell marker castor (red), nuclei counterstained with DAPI (blue) and phalloidin marking actin (green). White arrowheads point to stalk cells between stage 5/6 and 7/8 egg chambers. (H) Quantification of stalk cell numbers counted by castor^+^ cells between stage 5/6 and 7/8 egg chambers (arrowheads from images in E-G). Data are mean±s.e.m. from individual ovaries of at least nine animals (D) or from over 19 ovarioles from at least ten different animals (H). *P<0.01, **P<0.001, ***P<0.0001 (unpaired two-tailed Student's *t*-test).

To determine whether the arrested egg chambers underwent subsequent cell death, we immunostained ovaries with an antibody that detects proteolytically activated (cleaved) caspase Dcp-1 (Vasudevan and Ryoo, 2016). We observed that stage 7/8 egg chambers from several *crc^GFSTF^* and *crc^GFSTF^*/*crc^1^* ovarioles showed strong cleaved Dcp-1 staining (Fig. 7A-C). Analysis from over ten young animals (2 days old) indicated that at least one ovariole in each *crc* mutant ovary showed strong cleaved Dcp-1 staining in stage 7/8 egg chambers, in stark contrast to none in control ovaries (Fig. 7D). These data suggest that the decrease in fertility in *crc^GFSTF^* and *crc^GFSTF^*/*crc^1^* females is correlated with cell death in stage 7 and 8 egg chambers during oogenesis.

Reduced fertility has also been demonstrated to be a consequence of dysregulation of stalk cells, which connect the egg chambers of an ovariole (Fig. 6C; Borensztejn et al., 2018). Specifically, either a failure to reduce stalk cell numbers by apoptosis during development, or excessive culling of stalk cells, were shown to decrease fertility (Borensztejn et al., 2018). To examine whether the fertility defects in *crc* mutants could be attributed to dysregulation in stalk cell numbers, we stained control and mutant ovaries with a stalk cell marker, castor (Chang et al., 2013). We observed that although ovarioles from control egg chambers showed ∼7 stalk cells between their stage 5/6 and 7/8 egg chambers, *crc* mutants showed significantly fewer stalk cells (Fig. 7E-H). These data suggest that dysregulation of stalk cell apoptosis may contribute to the fertility defects seen in *crc* mutants.

To examine which cell types express Crc in the ovary, we immunostained ovaries with GFP antibody to detect Crc-GFP. However, we were unable to detect Crc-GFP with anti-GFP (Fig. S4B,C), suggesting that Crc may regulate ovulation non-autonomously. We also attempted western blotting of ovary extracts to detect Crc-GFP but did not observe any detectable signal (data not shown). A previous study had suggested a non-autonomous role for fat body *Gcn2* in the regulation of oogenesis (Armstrong et al., 2014). Consistent with these observations, we detected high levels of Crc-GFP fusion protein in adult abdominal fat tissues from *crc^GFSTF^* animals (Fig. S5A,B). Further, using a fat body-specific driver to deplete *crc* in adult fat tissues led to decreased egg laying (Fig. S5C), similar to the phenotypes observed in *crc* mutants (Fig. 6A). These data raise the possibility that Crc mediates Gcn2-signaling in fat tissues to non-autonomously regulate oogenesis.

## DISCUSSION

ISR signaling is associated with various pathological conditions, but the role of *Drosophila crc* in adult tissues had remained unclear. This may be partly because the cytogenetic location of *crc* is very close to FRT40, and therefore, attempts to study Crc function using conventional genetic mosaics have been unsuccessful. Our understanding of the role of Crc in adult *Drosophila* tissues thus far has entirely relied on RNAi experiments. However, loss-of-function mutants allow for unbiased discovery of developmental phenotypes, as exemplified in our present study, in which we examined the role of Crc in later developmental stages, adult tissues and during aging.

Generally, misfolding-prone membrane proteins, such as Rh1^G69D^, are thought to activate the PERK-mediated ISR response, among other ER stress responses (Donnelly et al., 2013). It is worth noting here that although both *Drosophila* and mouse models of adRP describe a protective role for *Perk* in retinal degeneration (Athanasiou et al., 2017; Chiang et al., 2012; Vasudevan et al., 2020), there has been conflicting evidence on the role of ATF4 in the mouse adRP model (Bhootada et al., 2016). In this study, we show that loss of *crc* accelerates the age-related retinal degeneration in a *Drosophila* model of adRP. As we have previously shown that *Perk* mutants similarly accelerate retinal degeneration in this model (Vasudevan et al., 2020), we interpret that *crc* mediates the effect of *Perk* in this model. Our data show that loss of Crc renders photoreceptors susceptible to age-related retinal degeneration in *ninaE* animals (solid red line, Fig. 4A). Along with our observation showing an increase in Crc protein levels in older flies (Fig. 4B,C), these data indicate that photoreceptors suffer from physiological stress which requires Crc for their proper function and survival during aging.

One of the visible phenotypes in adult *crc* mutants is ectopic wing venation (Fig. 5). It has previously been demonstrated that *Gcn2* depletion in the posterior compartment of imaginal discs results in ectopic wing vein formation, although RNAi experiments from this study indicated this phenotype to be *crc* independent (Malzer et al., 2018). This raises the possibility of insufficient *crc* suppression in this previous study. The study proposed that GCN2 regulates bone morphogenetic protein (BMP) signaling by modulating mRNA translation in wing discs via eIF2α phosphorylation and *Thor* induction. Our results are consistent with this proposal, as *Thor* is a transcription target of *crc.* In addition, we report here that *crc* loss affects wing size, a finding that has not been reported previously. Given that BMP signaling has also been extensively implicated in determining wing size (Gibson and Perrimon, 2005; Shen and Dahmann, 2005), it is possible that GCN2-Crc signaling regulates wing size via BMP signaling. It is equally possible that GCN2-Crc signaling affects tissue size through regulating amino acid transport and metabolism through autonomous and non-autonomous means.

Although wing development is not known to be sexually dimorphic, fat tissues are known to have sex-specific effects, with particularly profound effects on female fertility in flies and other sexually dimorphic organisms (Valencak et al., 2017). It has been previously demonstrated that loss of *crc* in *Drosophila* larvae leads to reduced fat content and increased starvation susceptibility (Seo et al., 2009). Hence, it is possible that the block in oogenesis in *crc* mutants (Figs 6, 7) is due to metabolic changes in the female fat body, although this remains to be directly tested. This hypothesis integrates well with our data showing high Crc activity in adult fat tissues (Fig. S5A,B) and with observations from a previous study that amino acid sensing by GCN2 in *Drosophila* adult adipocytes regulates germ stem cells in the ovary (Armstrong et al., 2014). Indeed, our preliminary analysis with fat body-specific depletion of *crc* using the 3.1Lsp2-Gal4 driver leads to reduced fertility and increased dorsal appendage defects, similar to those seen in *crc* mutants (Fig. S4C). Nonetheless, it remains possible that Crc acts autonomously in the ovary but is undetectable using our current methods (Fig. S4B,C). In summary, our study has found utilities for the *crc^GFSTF^* allele in discovering a new role for ISR signaling in disease models and during development, and also as an endogenous reporter for ISR activation.

## MATERIALS AND METHODS

Flies were reared on cornmeal-molasses medium at 25°C under standard conditions except for retinal degeneration experiments when they were reared under constant light. All fly genotypes and sources used in the study are listed in Table S1.

### Generation of UAS-eIF2α transgenic lines

Full-length *Drosophila eIF2α* cDNA was amplified from DGRC plasmid (clone LD21861) with EcoRI and XbaI restriction sites using the following primers: Fwd, 5′-GGAATTCATGGCCCTGACGTCGCGCTTCTAC-3′; and Rev, 5′-GCTCTAGACTAATCCTCTTCCTCCTCCTCATCCTC-3′. The resulting DNA fragment was cloned into the EcoRI and XbaI sites of pUAST-attB to generate pUAST-attB-eIF2α^WT^. For the eIF2α phosphorylation mutants, unique cut sites across the phosphorylation site were identified (AatII and AgeI), and the following gene fragments corresponding to S51A and S51D mutations were ordered from Integrated DNA Technologies (mutant residues underlined): eIF2α^S51A^ fragment, atggccctgacgtcgcgcttctacaacgagcggtatccggagatcgaggatgtcgttatggtgaacgtgctgtccatcgccgagatgggcgcctacgttcatctgcttgag tacaacaacatcgagggcatgatcctgctgtcggagctgGcccgccggcgcatccgctccatcaacaagctgattcgtgtcggcaagaccgaaccggtggtggtt; and eIF2α^S51D^ fragment, atggccctgacgtcgcgcttctacaacgagcggtatccggagatcgaggatgtcgttatggtgaacgtgctgtccatcgccgagatgggcgcctacgttcatctgcttgagtacaacaacatcgagggcatgatcctgctgtcggagctgGAccgccggcgcatccgctccatcaacaagctgattcgtgtcggcaagaccgaaccggtggtggtt. The pUAST-attB-eIF2α^WT^ plasmid described above was then restriction digested with AatII and AgeI, and the wild-type fragment was replaced with the synthetic mutant fragment (mutated nucleotides underlined) to generate pUAST-attB-eIF2α^S51A^ and pUAST-attB-eIF2α^S51D^. The plasmids were then targeted to the VK13 attP-9A landing site [Bloomington *Drosophila* Stock Center (BDSC), 9732] by BestGene Inc to generate transgenic lines that were placed in the same 76A2 genomic locus.

### Phenotype analysis

Lethal phase analysis was performed as described previously (Vasudevan et al., 2020). Right wings were severed from 1- to 4-day-old flies and imaged using a Nikon SMZ1500 microscope outfitted with a Nikon 8MP camera with NIS-Elements software. Wing size was measured using the regions of interest feature in ImageJ software.

Female fertility was quantified by placing five 1- to 4-day-old virgin females with five *yw* males (or *Dj-GFP* males for fertilization assays) in a vial containing standard medium enhanced with yeast to encourage egg laying. After allowing 1 day for acclimatization, the flies were moved to a new vial and the number of eggs laid in a 24-h period were counted and quantified. Eggs were imaged for Fig. 6B by placing them on an apple juice plate and capturing them with a Nikon SMZ1500 microscope outfitted with 8MP Nikon camera controlled by NIS elements software. Ovaries from female flies in this experiment were dissected in ice-cold PBS and similarly imaged on apple juice plates for Fig. S4A.

### qPCR analysis

Total RNA was prepared from five wandering third instar larvae using TriZol (Invitrogen), and cDNA was generated using random hexamers (Fisher Scientific) and Maxima H minus reverse transcriptase (Thermo Fisher Scientific) according to the manufacturer's protocol. qPCR was performed using PowerSYBR Green Mastermix (Thermo Fisher Scientific) using the following primers: *crc*: Fwd, 5′-GGAGTGGCTGTATGACGATAAC-3′, and Rev, 5′-CATCACTAAGCAACTGGAGAGAA5-3′; *Thor*: Fwd, 5′-TAAGATGTCCGCTTCACCCA-3′, and Rev, 5′-CGTAGATAAGTTTGGTGCCTCC-3′; and *Rpl15*: Fwd, 5′-AGGATGCACTTATGGCAAGC-3′, and Rev, 5′-CCGCAATCCAATACGAGTTC-3′.

### Immunostaining

Ovaries and fat bodies were dissected in ice-cold PBS from female flies reared for 2-3 days, along with *yw* males, on standard medium enhanced with dry yeast. Tissues were fixed in 4% paraformaldehyde (PFA) in PBS-Triton (0.2% Triton X-100 and 1× PBS) for 30 min, washed three times with PBS-Triton and blocked in PBS-Triton with 1% bovine serum albumin for 3 h (all at room temperature). Tissues were stained overnight at 4°C with the primary antibodies diluted in PBS-Triton, following which they were washed three times with PBS-Triton and incubated with Alexa Fluor-conjugated secondary antibodies (Invitrogen) in PBS-Triton for 3 h at room temperature. Tissues were mounted in 50% glycerol containing DAPI.

Eye imaginal discs were dissected from wandering third instar larva in ice-cold PBS and fixed in 4% PFA in PBS for 20 min, washed twice with PBS and permeabilized in 1× PBS-Triton for 20 min (all at room temperature). Discs were incubated in primary antibodies diluted in PBS-Triton for 2 h, washed three times in PBS-Triton, incubated in Alex aFluor-conjugated secondary antibodies (Invitrogen) in PBS-Triton for 1 h and washed three times in PBS-Triton, prior to mounting in 50% glycerol containing DAPI. Adult retinae were dissected and visualized with phalloidin as described previously (Huang et al., 2018). Antibodies used were as follows: phalloidin-Alexa 647 (1:1000, Invitrogen, A22287); chicken anti-GFP (1:500, Aves Labs, GFP-1020); rabbit anti-GFP (1:500, Invitrogen, A6455); rabbit anti-cleaved Dcp-1 (1:100, Cell Signaling Technology, 9578S); mouse anti-4C5 for Rh1 [1:500, Developmental Studies Hybridoma Bank (DSHB)]; rabbit anti-eIF2α (1:500, Abcam, ab5369); rabbit anti-S51 peIF2α (1:500, Abcam, ab32157); and rabbit anti-castor (1:50, gift from Dr Erika Bach, New York University Grossman School of Medicine). All images were obtained using a Zeiss LSM 700 confocal microscope with ZEN elements software and a 20× air or 40× water lens.

### Retinal degeneration

All experiments were performed in a *white* mutant background as *crc^GFSTF^*, *crc^1^* and *ninaE^G69D^* do not have eye color. Male flies 0-3 days old were placed (20 animals/vial) under 1000-lumen light intensity, and their pseudopupil structures (reflecting photoreceptor integrity) were monitored with a blue fluorescent lamp at 3-day intervals for a 30-day period. Medium was replaced every 3 days, and flies with disrupted pseudopupils in one or both eyes were marked as having retinal degeneration.

### Western blotting

Fly head extracts were prepared from six severed male fly heads in 30 µl lysis buffer containing 10 mM Tris HCl (pH 7.5), 150 mM NaCl, protease inhibitor cocktail (Roche), 1 mM EDTA and 1% SDS. Following SDS-PAGE and western blotting, proteins were detected using primary antibodies and IRDye-conjugated secondary antibodies (LI-COR) [primary rabbit anti-GFP (1:500, Invitrogen) and mouse anti-beta Tubulin (1:1000, DSHB)] on an Odyssey system.

### Amino acid deprivation

Female flies 0-3 days old were placed (10 animals/vial) in standard medium or in vials containing 5% sucrose and 2% agarose, prepared in distilled H_2_O. The number of survivors was counted every 24 h and survivors were moved to new medium.

### Quantification of data

The quantified values used for graphs are listed in Table S2.

## Supplementary Material

Supplementary information
