# Supplementary material for: A protein-trap allele reveals roles for Drosophila ATF4 in photoreceptor degeneration, oogenesis and wing development
Source: Dis Model Mech. 2022 Mar 16;15(3):dmm049119. doi: 10.1242/dmm.049119 (PMC8938396; doi:10.1242/dmm.049119)
Supplement: Supplementary information [file dmm-15-049119-s1.pdf]

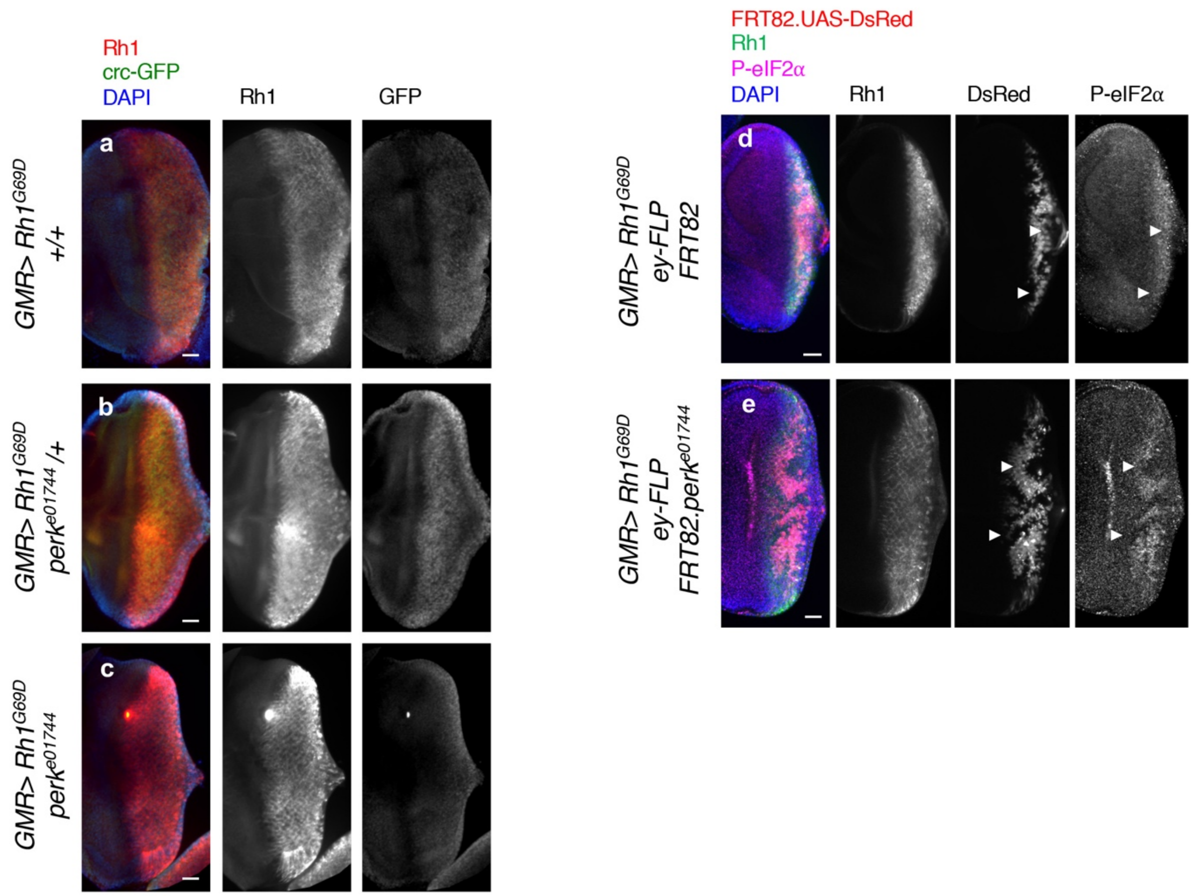

Fig. S1. crc-GFP induction in response to Rh1^{G69D} is PERK-dependent

a-c. Confocal images of third instar larval eye discs misexpressing mutant Rh1 (*GMR>Rh1^{G69D}*) in *Perk* +/+ control (a), *Perk^{e01744}*/+ heterozygous (b) or *Perk^{e01744}* homozygous animals (c) bearing one copy of *crc^{GFSTF}*. The left-most panels show merged images of crc-GFP (green) Rh1^{G69D} (red) and DAPI (blue). Rh1 and crc-GFP individual channels are also shown in black and white. d, e. Phospho-eIF2α staining (P-eIF2α, magenta) in *GMR>Rh1^{G69D}* eye discs containing *Perk^{e01744}* homozygous mosaic clones (e), or with control clones (d). *Perk^{e01744}* homozygous clones are marked by the absence of DsRed (red). DAPI (blue) counterstains the nucleus. Grayscale images of Rh1, DsRed and P-eIF2α only channels are shown in separate panels. Scale bars represent 25 μM.

Disease Models & Mechanisms • Supplementary information

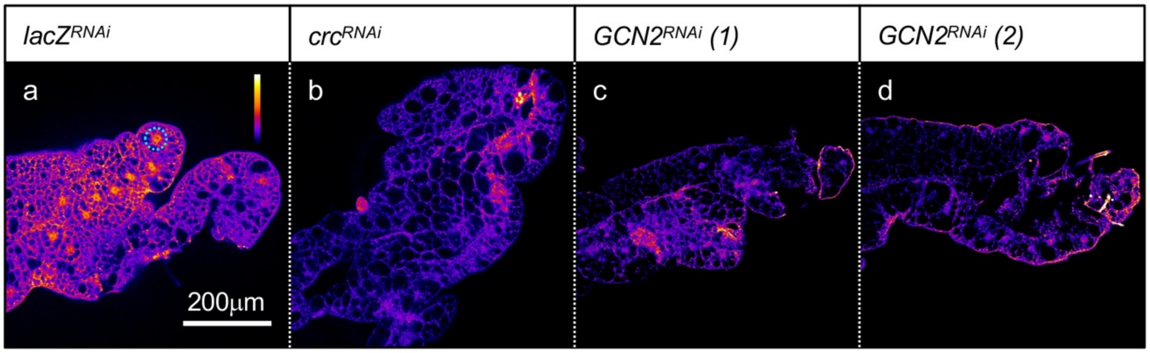

Fig. S2. crc-GFP expression in larval fat body is GCN2-dependent

(a-d). Fat body tissues dissected from posterior regions of male third instar larva shows strong nuclear expression of crc-GFP as seen by a heatmap of GFP signal (a). A representative nucleus is marked with a dotted circle. RNAi depletion of *crc* with fat body-specific driver *dcg-Gal4* shows loss of this signal (b), corroborating the specificity of immunostaining. Depletion of *GCN2* with two independent RNAi lines shows loss of nuclear crc-GFP signal (c, d).

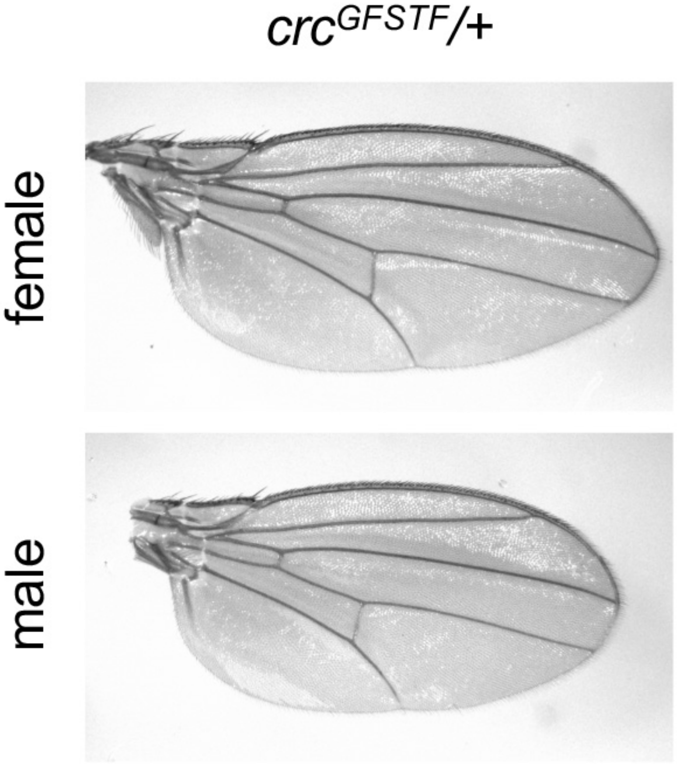

Fig. S3. ***crc*^{GFSTF} does not have a dominant effect**

Grayscale images of the right wing from female or male *crc*^{GFSTF}/+ heterozygotes do not show the wing defects seen in *crc* mutants (Fig. 2b-c).

Disease Models & Mechanisms • Supplementary information

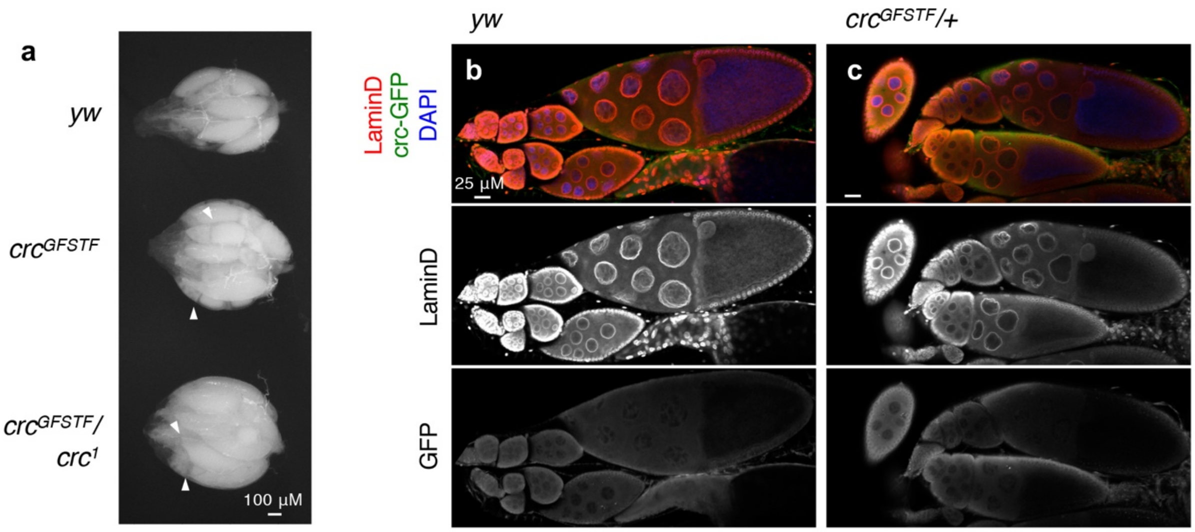

Fig. S4. crc reporters are not expressed in the ovary

a. Brightfield grayscale image of whole ovaries from indicated genotypes, with the germarium on the left (anterior) and oviduct to the right (posterior). White arrowheads point to easily discernible enlarged stage 10 egg chambers in intact ovarioles from *crc* mutants (*crc*^{GFSTF} and *crc*^{GFSTF}/*crc*^{1}).

b-c. Confocal images of ovarioles dissected from control (*yw*, b), and *crc*^{GFSTF}/+ animals (c) stained with anti-GFP to detect crc-GFP (green) and nuclei counterstained with DAPI (blue). Since DAPI staining is not readily visible in the oocyte, ovaries were also stained with lamin to visualize the nuclear envelope (red). Note that no significant crc-GFP signal was detected in *crc*^{GFSTF} in comparison to *yw*.

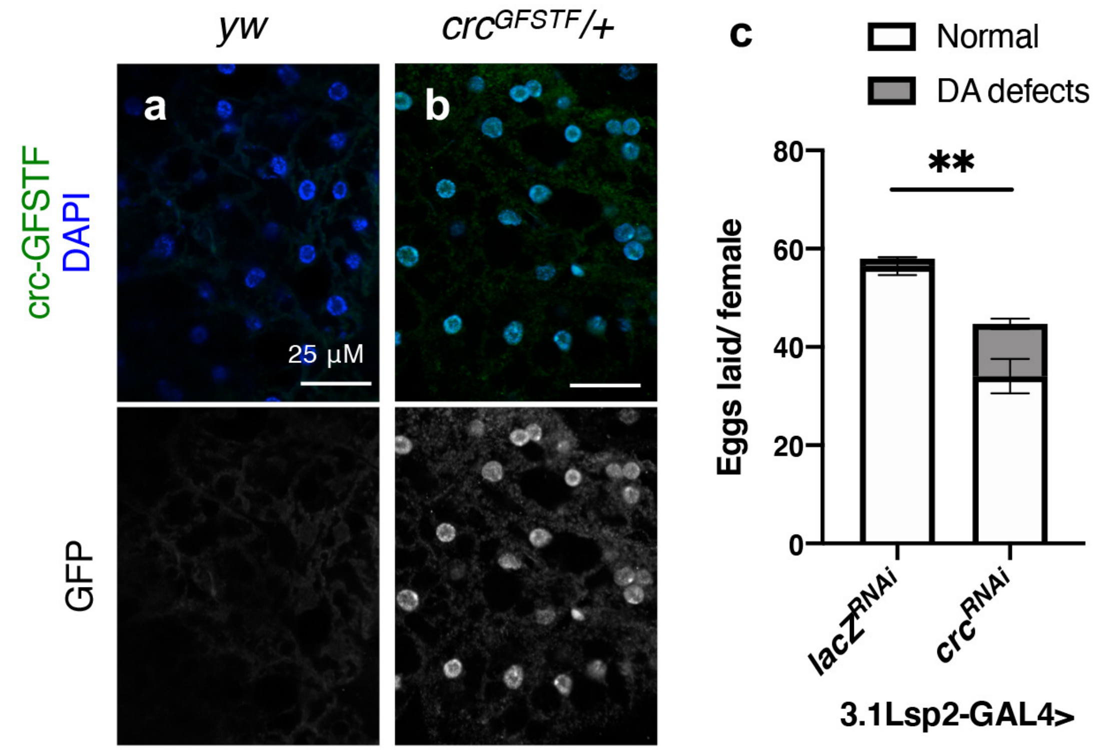

Fig. S5. crc-GFP is expressed in the fat body and can regulate fertility

a, b. Confocal images of adult fat bodies from animals of indicated genotypes stained with anti-GFP (green) and nuclei counterstained with DAPI (blue), showing robust expression of crc-GFP in adipocyte cells.

c. Total number of eggs laid per female in a 24-hour period for control (*lacZ*^{RNAi}) and *crc*^{RNAi} driven by fat body specific 3.1Lsp2-Gal4, with proportion of eggs showing dorsal appendage (DA) defects in gray. The data are the mean from 3 independent experiments with four females per experiment, error bars represent standard error. **= $p<0.001$, calculated by a paired two-tailed *t*-test.

Table S1. Stocks used in the study and their source.

Genotype	Source/reference
yw	BDSC 1495
crc^{GFSTF}	BDSC 59608
crc^{1}	BDSC 266
crc^{R6}	(Hewes et al., 2000)
Df(2L)Exel7081	BDSC 7855
Dp(90599)	BDSC 90599
$ninaE^{G69D}$	BDSC 64123
$UAS\text{-}Rh1^{G69D}$	(Kang and Ryoo 2009)
$perk^{e01744}$	BDSC 85557
$4E\text{-}BP^{intron}\text{-}DsRed$	(Kang et al. 2017)
Dj-GFP	BDSC 5417
3.1Lsp2-Gal4	(Armstrong et al., 2014)
$lacZ^{RNAi}$	(Kang et al., 2017)
crc^{RNAi}	(Kang et al., 2017)
$GCN2^{RNAi}$ *(1)*	VDRC 103976
$GCN2^{RNAi}$ *(2)*	HMC06316

Table S2. Quantification values for graphs in figures.

Click here to download Table S2
